# Pretreatment Lactate Dehydrogenase-to-Albumin Ratio Identifies an Early Adverse Prognostic Profile in IDH-Wildtype Glioblastoma: A Real-World Cohort Study

**DOI:** 10.3390/medicina62101958

**Published:** 2026-10-10

**Authors:** Hayrani Kaya, Asim Armagan Aydin, Abdullah Umit, Ozan Deniz Guven, Ahmet Unlu, Fahriye Merve Balcioglu, Banu Ozturk, Mustafa Yildiz, Murat Kocer

**Affiliations:** Department of Clinical Oncology, Antalya Training and Research Hospital, University of Health Sciences, 07100 Antalya, Turkey; docabdullah036@gmail.com (A.U.); odg295@gmail.com (O.D.G.); md.ahmetunlu@gmail.com (A.U.); fmerbalcioglu@gmail.com (F.M.B.); drbanutr@yahoo.com (B.O.); drmyildiz@yahoo.com (M.Y.); muratkocer71@hotmail.com (M.K.)

**Keywords:** glioblastoma, IDH-wildtype, diffuse glioma, CNS WHO grade 4, lactate dehydrogenase-to-albumin ratio, systemic inflammation, prognostic biomarker, risk stratification

## Abstract

*Background and Objectives*: Clinical outcomes remain markedly heterogeneous in glioblastoma, isocitrate dehydrogenase (IDH)-wildtype, despite contemporary molecular classification and established clinicopathological risk factors. We investigated whether the pretreatment lactate dehydrogenase-to-albumin ratio (LAR), an accessible circulating index integrating systemic disease- and host-related information, provides complementary prognostic information. *Materials and Methods*: We retrospectively evaluated 134 patients with glioblastoma, IDH-wildtype, central nervous system (CNS) World Health Organization (WHO) grade 4. An exploratory, cohort-specific LAR threshold was derived for 12-month overall survival (OS) and retained without re-optimization across subsequent analyses. Associations with OS and progression-free survival (PFS) were evaluated using Cox regression, and incremental prognostic performance was assessed against a baseline clinicopathological model. Temporal and continuous associations with survival, internal model validation, and the relationship between LAR and treatment response during adjuvant temozolomide therapy were also examined. *Results*: The cohort-derived LAR threshold was 5.718. High pretreatment LAR was associated with shorter median OS (5.5 vs. 16.0 months) and PFS (5.2 vs. 10.6 months; both *p* < 0.001) and remained independently associated with OS (adjusted hazard ratio [HR], 4.51; 95% CI, 2.84–7.18) and PFS (adjusted HR, 3.60; 95% CI, 2.33–5.56). Adding LAR to the clinicopathological model increased Harrell’s C-index from 0.699 to 0.760 for OS and from 0.690 to 0.759 for PFS (both likelihood ratios, *p* < 0.001), with optimism-corrected C-indices of 0.743 and 0.742, respectively. Prognostic discrimination and adjusted effect estimates were greatest early in the follow-up period and attenuated over time, whereas continuous LAR showed nonlinear associations with both endpoints. Among the 104 patients who received adjuvant temozolomide, a high LAR was independently associated with progressive disease (adjusted odds ratio, 3.72; 95% CI, 1.28–10.81). *Conclusions*: Pretreatment LAR identified patients with IDH-wildtype glioblastoma who experienced poorer survival, and the prognostic effect was concentrated predominantly in the early disease course. Its incremental contribution beyond conventional clinicopathological characteristics supports the further evaluation of LAR as an accessible component of baseline risk characterization. However, independent external validation is required before clinical application.

## 1. Introduction

Glioblastoma, isocitrate dehydrogenase (IDH)-wildtype, central nervous system (CNS) World Health Organization (WHO) grade 4, is the most aggressive adult-type diffuse glioma, characterized by rapid progression, near-universal recurrence, and persistently poor survival despite intensive multimodal therapy [1,2,3]. Although advances in surgical and radiotherapeutic techniques and the development of immune-based, cellular, molecularly targeted, and oncolytic strategies have broadened the therapeutic landscape [4,5], durable disease control remains uncommon and meaningful improvements in population-level survival have been limited [6]. In parallel, contemporary molecular classification has substantially refined the biological definition of adult-type diffuse gliomas [7]; however, marked heterogeneity in clinical outcomes persists even within the molecularly defined entity of glioblastoma, IDH-wildtype [8]. Age, performance status, extent of resection, and O6-methylguanine-DNA methyltransferase (MGMT) promoter methylation remain central to prognostic assessment; however, substantial outcome variability persists after these factors are considered. This residual heterogeneity provides a rationale for complementary markers capable of adding information to the baseline risk characterization.

Routine blood measurements provide an accessible means of capturing systemic features that may accompany glioma progression. Composite inflammatory indices, including the neutrophil-to-lymphocyte ratio (NLR) [9], systemic immune-inflammation index (SII) [10], and systemic inflammation response index (SIRI) [11], have consequently attracted interest as prognostic markers in glioma, although their associations with clinical outcomes have varied across studies. These indices primarily characterize relationships among circulating immune and inflammatory cell populations. Biochemical parameters reflecting other aspects of the systemic response to malignancy may provide distinct and potentially complementary prognostic information. In particular, measures associated with tissue or metabolic stress and with the physiological condition of the host have received comparatively limited attention in molecularly defined glioblastoma.

The lactate dehydrogenase-to-albumin ratio (LAR) combines two routinely measured circulating parameters that reflect distinct aspects of systemic physiology. Lactate dehydrogenase (LDH) may increase in the setting of cellular turnover, tissue injury, hypoxic stress, and altered energy metabolism [12,13]. As a key enzyme in lactate–pyruvate metabolism, LDH is closely linked to metabolic adaptation under conditions of increased energy demand and limited oxygen availability [14,15]. Albumin, by contrast, is shaped by inflammatory activity, hepatic synthesis, nutritional status, and overall physiological reserve [16]. Declining albumin concentrations may therefore accompany the systemic consequences of inflammation, catabolic stress, and impaired host condition in patients with cancer [17]. Together, these biologically distinct components provide a rationale for examining LAR as a composite marker potentially reflecting both disease-related stress and host physiological state. Across several solid malignancies, higher LAR has been associated with unfavorable outcomes [18,19,20], suggesting that the ratio may carry prognostic information beyond that conveyed by either component alone. This concept is particularly relevant to glioblastoma, where metabolic plasticity, regional hypoxia, and dynamic interactions between malignant and myeloid cell populations contribute to disease progression and therapeutic resistance [21]. Emerging experimental evidence linking LDHA-dependent metabolism and lactate signaling to tumor–myeloid interactions provides further biological rationale for investigating an LDH-containing circulating index in this setting []. Nevertheless, circulating LAR—and serum total LDH specifically—cannot be regarded as a direct measure of tumor LDHA expression or activity, glycolytic flux, intratumoral lactate production, hypoxia, or immune activity, and its clinical significance in IDH-wildtype glioblastoma remains insufficiently defined.

Although LAR is derived from routinely available laboratory measurements, its prognostic relevance has not been comprehensively characterized in molecularly defined IDH-wildtype glioblastoma, and its incremental contribution beyond established clinicopathological factors remains uncertain. Against this background, we investigated the prognostic significance of pretreatment LAR in a real-world cohort of patients with glioblastoma, IDH-wildtype, CNS WHO grade 4. We examined its association with overall and progression-free survival and whether it provided prognostic information beyond established clinicopathological characteristics and circulating inflammatory indices. We further characterized the temporal and continuous nature of its association with survival and examined its relationship with treatment response during adjuvant temozolomide. Together, these analyses were designed to determine whether pretreatment LAR captures clinically relevant prognostic information that may complement conventional baseline risk assessment in IDH-wildtype glioblastoma.

## 2. Materials and Methods

### 2.1. Study Design and Population

We conducted a retrospective, single-center cohort study of patients managed for glioblastoma, IDH-wildtype, CNS WHO grade 4, at the Department of Clinical Oncology, University of Health Sciences Antalya Training and Research Hospital from October 2014 through April 2024. Diagnostic classification was retrospectively aligned with the 2021 World Health Organization Classification of Tumours of the Central Nervous System (WHO CNS5) [7] using histopathological and molecular information available for each patient. The analytic cohort comprised patients who met the diagnostic definition of glioblastoma, IDH-wildtype, and CNS WHO grade 4, together with the predefined clinical, treatment, and laboratory eligibility requirements.

A total of 242 patients with grade 4 glioma were screened for eligibility. Patients were excluded for the following reasons: IDH-mutant disease (*n* = 46), absence of surgical resection (*n* = 14), active infection requiring antibiotic therapy (*n* = 6), chronic inflammatory disease requiring systemic corticosteroid treatment (*n* = 8), blood transfusion or albumin replacement within 1 month before pretreatment laboratory assessment (*n* = 4), inability to receive standard radiotherapy (*n* = 11), or insufficient clinical or laboratory data for the planned analyses (*n* = 19). After applying these criteria, 134 patients with glioblastoma, IDH-wildtype, CNS WHO grade 4, constituted the final analytic cohort. The patient selection process is shown in Figure 1.

Clinical, pathological, molecular, laboratory, treatment, imaging, and survival data were retrospectively extracted from institutional medical records. The Institutional Review Board of the University of Health Sciences Antalya Training and Research Hospital approved the study protocol (Approval No. 2026-26; Decision No. 1/7; 8 January 2026). All study procedures conformed to the principles of the Declaration of Helsinki. Because the study relied exclusively on retrospective data, the Institutional Review Board waived the requirement for informed consent.

### 2.2. Clinical, Pathological and Treatment Characteristics

Baseline demographic and clinical variables included age at diagnosis, sex, comorbidity status, smoking history, body mass index (BMI), Eastern Cooperative Oncology Group (ECOG) performance status, and the presence of neurological deficits or epileptic seizures at presentation. Tumor-related characteristics included anatomical location, lateralization, focality, maximum tumor diameter, extent of resection, Ki-67 labeling index, MGMT promoter methylation status, and alpha-thalassemia/mental retardation syndrome X-linked (ATRX) status, where available.

Postoperative clinical and radiological documentation was used to classify the extent of resection as gross total resection (GTR) or non-GTR. MGMT promoter methylation status was recorded as methylated, unmethylated, or unknown. Testing was not consistently available over the entire study period; consequently, missing MGMT results were not imputed and were modeled as unknown categories in the primary multivariable analyses.

Postoperative treatment was delivered within a Stupp-based framework [22]. Radiotherapy consisted of conventional fractionation to 60 Gy in 30 fractions in all patients. Concurrent and subsequent temozolomide was prescribed according to standard institutional practice and individual clinical suitability. The receipt of adjuvant temozolomide and its total duration were recorded separately. Because treatment duration is determined after the baseline assessment, it was treated as a post-baseline exposure and excluded from the primary pretreatment prognostic models.

### 2.3. Pretreatment Laboratory Assessment and Biomarker Calculation

Laboratory measurements used to derive the study biomarkers were obtained from peripheral blood and serum collected within 7 days before surgical resection and before any oncological treatment. Available measurements included serum LDH, albumin, C-reactive protein (CRP), absolute neutrophil, lymphocyte, monocyte, and platelet counts. Albumin measurements were standardized to g/L across the study period before calculating the derived indices, while CRP was expressed in mg/L. All components entering a given index originated from the same pretreatment sampling time point.

The primary biomarker of interest was the lactate dehydrogenase-to-albumin ratio (LAR) [20], calculated as the serum LDH divided by the serum albumin. For comparative analyses, six established systemic inflammatory or inflammation-related composite indices were additionally derived: the neutrophil-to-lymphocyte ratio (NLR; neutrophils/lymphocytes) [9], systemic immune-inflammation index (SII; platelets × neutrophils/lymphocytes) [10], systemic inflammation response index (SIRI; neutrophils × monocytes/lymphocytes) [11], pan-immune-inflammation value (PIV; neutrophils × platelets × monocytes/lymphocytes) [23], C-reactive protein-to-albumin ratio (CAR; CRP/albumin) [24], and monocyte-to-albumin ratio (MAR; monocytes/albumin) [25].

### 2.4. Radiological Assessment and Treatment Response

Serial contrast-enhanced magnetic resonance imaging (MRI) obtained as part of routine follow-up formed the basis of the radiological assessment. Response and progression were determined according to the Response Assessment in Neuro-Oncology (RANO) [26] principles, with imaging interpreted alongside the available clinical information.

For response analysis, treatment outcomes during adjuvant temozolomide were classified as disease control or progressive disease (PD) based on documented clinical and radiological assessments. Disease control encompassed complete response (CR) and non-progressive residual disease, whereas PD represented an adverse response category. The analysis was restricted to patients who received adjuvant temozolomide and had evaluable treatment responses.

### 2.5. Study Endpoints

The primary endpoint was overall survival (OS), defined as the interval from the date of pathological diagnosis to death from any cause. Patients alive at the last documented follow-up were censored at that date.

Progression-free survival (PFS) was evaluated as a secondary endpoint and defined as the interval from the date of pathological diagnosis to radiologically documented disease progression or death from any cause, whichever occurred first. Patients without a PFS event were censored on the date of their last disease assessment.

The association between pretreatment LAR and treatment response during adjuvant temozolomide was evaluated as an additional clinical outcome using the disease control versus progressive disease classification defined above.

### 2.6. Statistical Analysis

Descriptive summaries are reported as medians (IQR) for continuous measures and as counts with percentages for categorical measures. Group differences were examined using the Mann–Whitney U test for continuous variables and the χ^2^ or Fisher’s exact test for categorical variables, as appropriate. The follow-up duration was characterized using the reverse Kaplan–Meier estimator.

The prognostic discrimination of pretreatment LAR was evaluated primarily for 12-month OS using receiver operating characteristic (ROC) analysis. The Youden index identified an exploratory, cohort-specific threshold of 5.718, which was retained without re-optimization for all subsequent categorical analyses. Bootstrap resampling (1000 iterations) was used to assess the internal stability of the threshold, rather than to establish external validity. Inverse probability of censoring weighting (IPCW)-adjusted time-dependent ROC analyses were used to characterize discrimination over time for OS and PFS. LAR was compared with NLR, SII, SIRI, PIV, CAR, and MAR using paired DeLong tests, with Holm correction for the six comparisons.

Kaplan–Meier estimates were used to describe survival distributions, and between-group differences were evaluated using the log-rank test. Cox proportional hazards models were used to quantify the associations with OS and PFS, yielding hazard ratios (HRs) and 95% confidence intervals (CIs). The baseline multivariable specification for each endpoint comprised age, ECOG performance status, MGMT promoter methylation status, multifocal disease, extent of resection, and Ki-67 labeling index; LAR status was then added to construct an augmented model. To assess the potential influence of missing MGMT promoter methylation data, a sensitivity analysis was performed by repeating the augmented multivariable Cox models after restricting the cohort to patients with known MGMT status (methylated or unmethylated). Variables arising after baseline, including adjuvant temozolomide duration and treatment response, were deliberately excluded. Model improvement after the addition of LAR was evaluated using the Akaike information criterion (AIC), Harrell’s C-index, and likelihood ratio testing.

Subgroup-specific Cox models and interaction terms were used to assess consistency and effect modification across age, sex, ECOG performance status, extent of resection, tumor focality, tumor size, ATRX status, and Ki-67 labeling index. Landmark analyses were performed at 3 and 6 months, requiring patients to be alive for OS analyses and alive and progression-free for PFS analyses at the corresponding landmarks. The association between LAR and treatment response during adjuvant temozolomide was evaluated using Mann–Whitney U and Fisher’s exact tests and binary logistic regression analysis. The incremental performance of the augmented logistic model was assessed using AIC, ROC AUC, McFadden pseudo-R^2^, and likelihood ratio testing.

Internal validation of the augmented Cox models used 500 bootstrap resamples to estimate the optimism-corrected Harrell C-indices. The proportional hazards assumption was assessed using scaled Schoenfeld residuals, with complementary time-varying Cox analyses used to characterize the temporal variation in the LAR effect. Because dichotomization may obscure information contained in a continuous biomarker, categorical analyses were used primarily to provide an interpretable exploratory risk-stratification framework and to facilitate Kaplan–Meier visualization, subgroup, landmark, and treatment-response analyses. The underlying association between LAR and survival was therefore also characterized on its continuous scale. Continuous associations between LAR and survival were examined using restricted cubic spline models adjusted for the same clinicopathological covariates, with LAR of 5.718 as the reference value.

Analyses were performed using R version 4.4.1 (R Foundation for Statistical Computing, Vienna, Austria) and IBM SPSS Statistics for Windows version 27.0 (IBM Corp., Armonk, NY, USA). All tests were two-sided, with *p* < 0.05 considered statistically significant, unless multiplicity-adjusted *p* values were reported.

## 3. Results

### 3.1. Cohort Characteristics

A total of 134 patients with glioblastoma, IDH-wildtype, CNS WHO grade 4, who fulfilled the predefined eligibility criteria constituted the final analytic cohort (Table 1). Using the cohort-derived pretreatment LAR cutoff value of 5.718, 77 patients (57.5%) were classified as LAR-low and 57 (42.5%) as LAR-high.

The median age of the overall cohort was 60.0 years (interquartile range [IQR], 52.2–67.0), and 74 patients (55.2%) were men. Most patients had an ECOG performance status of 0–1 (72.4%), while neurological deficits and epileptic seizures at presentation were observed in 20.9% and 26.1% of the patients, respectively. GTR was achieved in 62 patients (46.3%), and multifocal disease was present in 33 patients (24.6%). The median tumor diameter was 44.0 mm (IQR, 35.0–53.0 mm), and the median Ki-67 labeling index was 40.0% (IQR, 20.0–60.0). Overall, 104 patients (77.6%) received adjuvant temozolomide, with a median treatment duration of 6.0 months (IQR, 6.0–8.0).

A comparison of the baseline characteristics between the LAR groups identified several clinically meaningful differences (Table 1). Patients with elevated pretreatment LAR had a lower body mass index than those in the LAR-low group (median, 24.2 vs. 28.1 kg/m^2^; *p* < 0.001). Differences in subsequent treatment exposure were also evident, with patients in the LAR-high group being less likely to receive adjuvant temozolomide and, among treated patients, having a shorter duration of therapy (both *p* < 0.001).

In addition, the baseline demographic, clinical, and tumor-related characteristics were generally balanced between the two groups (Table 1).

At a median follow-up of 34.4 months (95% CI, 23.8 months to not reached), estimated using the reverse Kaplan–Meier method, 127 patients (94.8%) experienced disease progression, and 124 (92.5%) died. The median OS and PFS for the overall cohort were 10.6 months (95% CI, 9.6–13.2) and 8.7 months (95% CI, 7.0–10.3), respectively.

### 3.2. Prognostic Discrimination and Threshold Derivation of Pretreatment Lactate Dehydrogenase-to-Albumin Ratio

The prognostic discrimination of the pretreatment LAR was primarily evaluated at the clinically relevant 12-month OS time point. Conventional ROC analysis was used to derive an exploratory, cohort-specific risk-stratification threshold, while IPCW-adjusted time-dependent ROC analyses were performed to account for censoring and to characterize discrimination across clinically relevant follow-up intervals. The performance of LAR was compared with that of established systemic inflammatory indices.

For the 12-month OS, the pretreatment LAR yielded an area under the ROC curve (AUC) of 0.782. The Youden-derived threshold was 5.718, corresponding to a sensitivity of 68.0% and specificity of 89.5%. This exploratory cohort-derived threshold was retained for all subsequent categorical Kaplan–Meier, Cox regression, subgroup, landmark, and treatment response analyses. When the same threshold was applied to the 6-month PFS, LAR yielded an AUC of 0.758, with a sensitivity of 68.8% and specificity of 73.9% (Figure 2).

**Figure 2:**
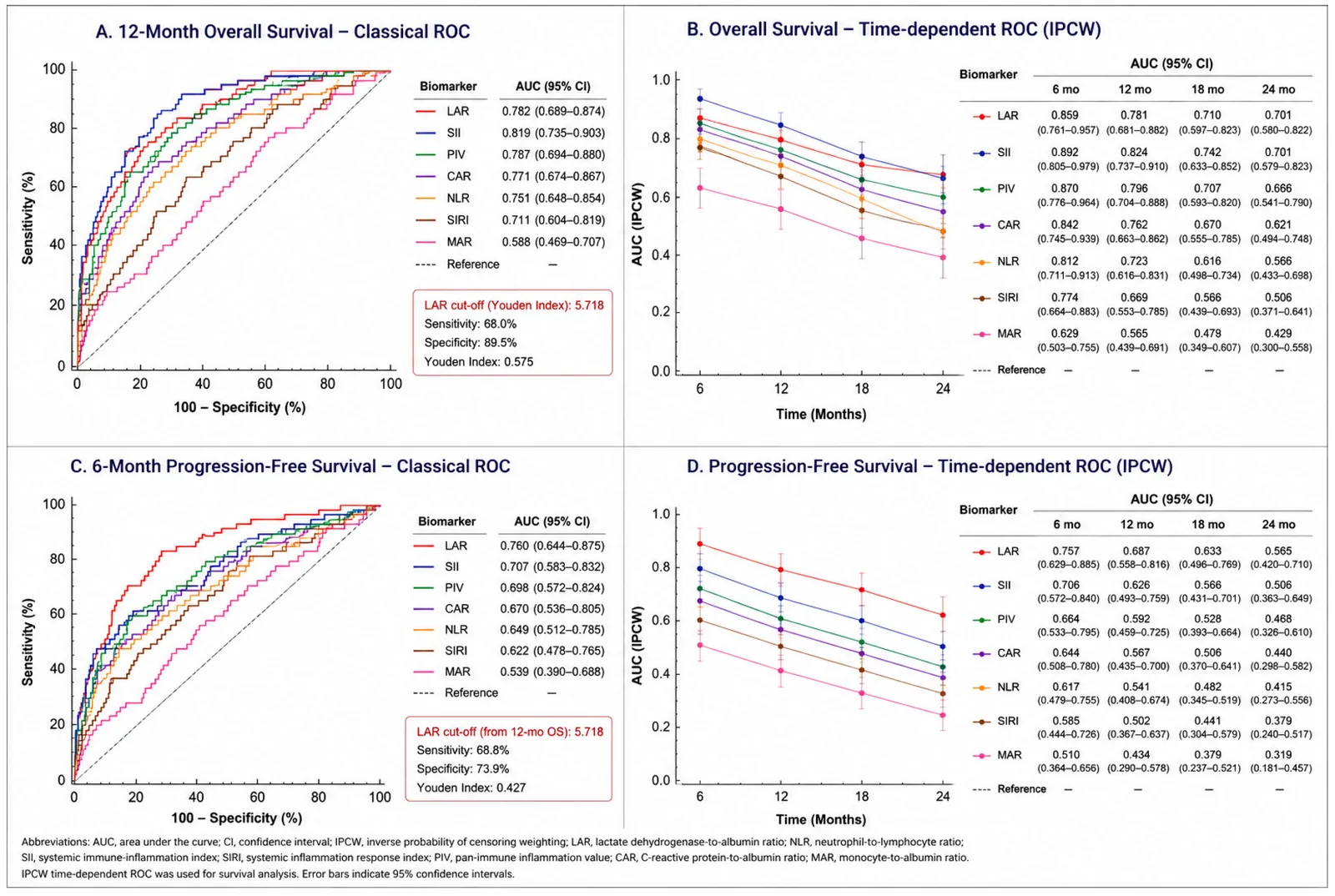
Prognostic discrimination of pretreatment LAR and comparator systemic inflammatory biomarkers. Footnote: Conventional ROC analyses for 12-month overall survival (**A**) and 6-month progression-free survival (**C**), together with IPCW-adjusted time-dependent ROC analyses for overall survival (**B**) and progression-free survival (**D**). The clinically relevant 12-month OS time point was used to derive the exploratory, cohort-specific LAR threshold of 5.718 using the Youden index. The same threshold was retained without re-optimization for all subsequent categorical analyses.

Bootstrap resampling with 1000 iterations supported the internal stability of the selected threshold. The median bootstrap-derived optimal LAR threshold was identical to the original cohort estimate at 5.718, with a 95% bootstrap interval of 5.47–5.93. The mean bootstrap AUC was 0.783 (95% bootstrap interval, 0.704–0.863), and the median bootstrap sensitivity and specificity were 68.8% and 89.6%, respectively. These findings indicate internal stability of the cohort-derived estimate around 5.7 but should not be interpreted as external validation of the threshold, which remains exploratory and requires independent evaluation in external cohorts (Figure 3).

**Figure 3:**
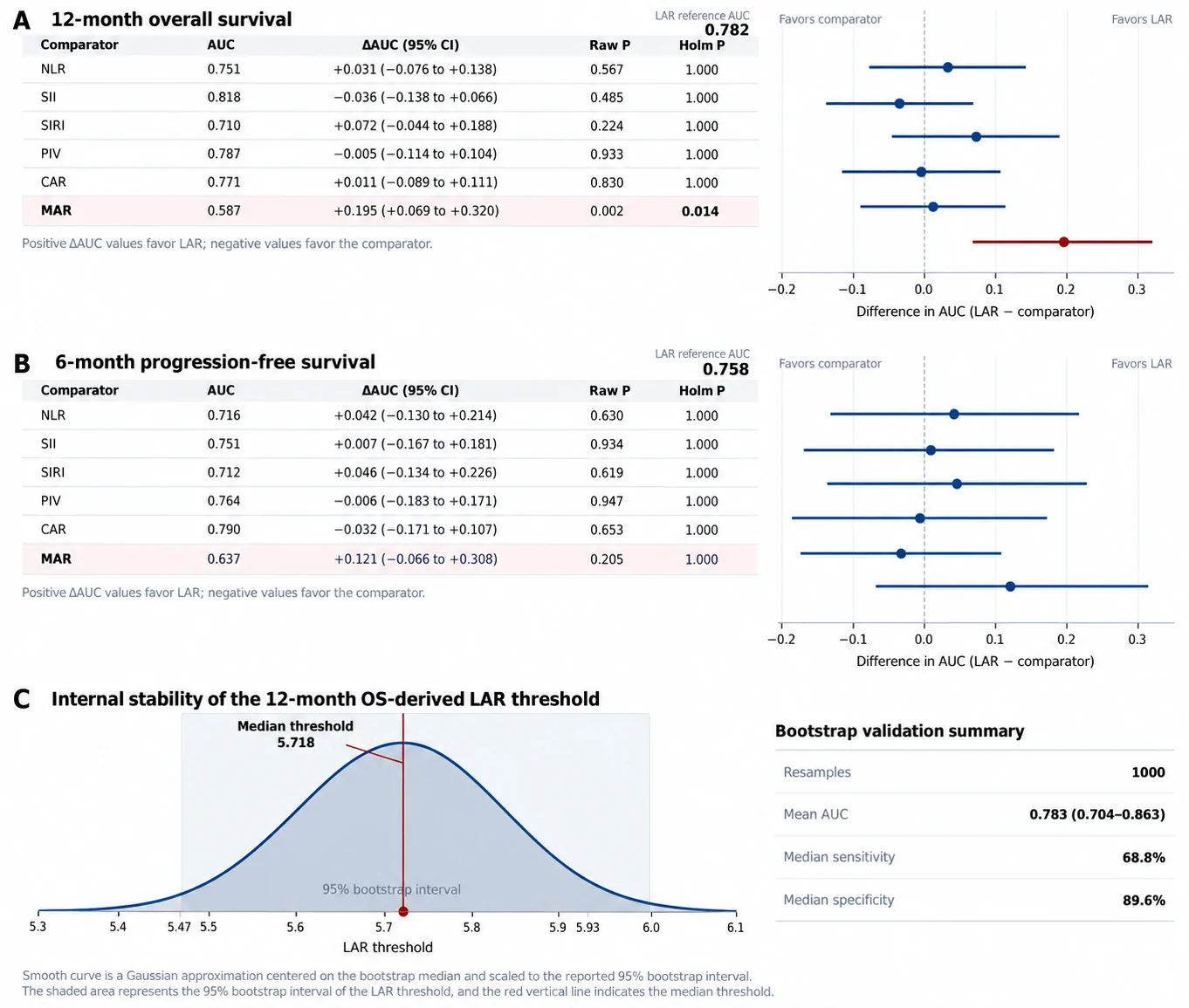
Comparative prognostic discrimination and internal validation of the pretreatment LAR. Footnote: Panels (**A**,**B**) compare the discriminatory performance of pretreatment LAR with that of each comparator inflammatory biomarker for predicting 12-month OS and 6-month PFS, respectively. Positive ΔAUC values indicate greater discriminatory performance of LAR than the comparator biomarker. Error bars represent the 95% confidence intervals derived from the paired DeLong analyses. Raw and Holm-adjusted *p* values are reported for the six LAR-versus-comparator comparisons.In panels (**A**,**B**), the light pink background highlights the MAR comparison row. Blue circles and horizontal lines indicate the AUC differences and their 95% confidence intervals for the other comparator biomarkers. In panel (**A**), the dark red circle and horizontal line identify the MAR comparison, which remained statistically significant after Holm correction; in panel (**B**), the MAR comparison is displayed in blue because it was not statistically significant after correction. The vertical dashed lines in both panels indicate no difference in AUC (ΔAUC = 0).Panel (**C**) summarizes bootstrap assessment of the internal stability of the exploratory Youden-derived LAR threshold using 1000 resamples, showing the median bootstrap-derived threshold and its 95% bootstrap interval. The cohort-derived threshold was 5.718. The light-colored rectangular background indicates the 95% bootstrap interval of the threshold (5.47–5.93), whereas the red vertical line and circle mark the median bootstrap-derived threshold. The smooth curve represents a Gaussian approximation centered on the bootstrap median and scaled to the reported bootstrap interval. Abbreviations: AUC, area under the receiver operating characteristic curve; CAR, C-reactive protein-to-albumin ratio; MAR, monocyte-to-albumin ratio; NLR, neutrophil-to-lymphocyte ratio; OS, overall survival; PFS, progression-free survival; PIV, pan-immune-inflammation value; SII, systemic immune-inflammation index; SIRI, systemic inflammation response index.

Time-dependent analyses demonstrated that the prognostic discrimination of LAR was strongest during the early disease course and progressively decreased with longer follow-up. The IPCW-adjusted AUCs for OS were 0.859, 0.781, 0.710, and 0.701 at 6, 12, 18, and 24 months, respectively. The corresponding AUCs for PFS were 0.757, 0.687, 0.633, and 0.565, respectively. Thus, pretreatment LAR showed the greatest discriminatory performance for early mortality and disease progression, with more modest discrimination at later time points (Figure 2).

In pairwise DeLong comparisons, the discriminatory performance of LAR for 12-month OS was comparable to that of NLR, SII, SIRI, PIV, and CAR. The LAR demonstrated greater discrimination than MAR (ΔAUC, 0.195; unadjusted *p* = 0.002), and this difference remained statistically significant after Holm correction for the six LAR-versus-comparator comparisons (adjusted *p* = 0.014). No multiplicity-adjusted differences were observed between LAR and the remaining inflammatory indices. For 6-month PFS, none of the comparator biomarkers differed significantly from the LAR before or after Holm’s adjustment (Figure 3).

### 3.3. Survival According to Pretreatment Lactate Dehydrogenase-to-Albumin Ratio

Kaplan–Meier analyses of OS and PFS according to the pretreatment LAR status are presented in Figure 4. The median OS was 16.0 months (95% confidence interval [CI], 12.5–21.6) in the LAR-low group and 5.5 months (95% CI, 4.0–7.3) in the LAR-high group. A high pretreatment LAR was associated with a shorter OS (hazard ratio [HR], 4.02; 95% CI, 2.63–6.15; log-rank *p* < 0.001) (Figure 4A). The median PFS was 10.6 months (95% CI, 8.2–13.8) in the LAR-low group and 5.2 months (95% CI, 3.7–6.6) in the LAR-high group. A high pretreatment LAR was also associated with shorter PFS (HR, 3.85; 95% CI, 2.40–6.16; log-rank *p* < 0.001) (Figure 4B).

### 3.4. Cox Proportional Hazards Regression Analyses

Univariable Cox proportional hazards analyses identified several clinicopathological and inflammatory variables associated with both OS and PFS (Table 2). Poor ECOG performance status, multifocal disease, non-GTR, unmethylated MGMT promoter status, higher Ki-67 labeling index, shorter adjuvant temozolomide duration, and elevated pretreatment inflammatory biomarkers—including LAR, NLR, SII, SIRI, PIV, CAR, and MAR—were significantly associated with inferior survival outcomes. In contrast, age, sex, comorbidity, neurological deficit, epileptic seizure, tumor size, and tumor location were not significantly associated with either endpoint. Although adjuvant temozolomide duration demonstrated a significant association with both OS and PFS in univariable analyses, it was not incorporated into the primary multivariable models because it represents a post-baseline treatment variable rather than a baseline prognostic characteristic. Similarly, radiological treatment response was not considered for multivariable modelling because it reflects a post-treatment outcome rather than a pre-treatment prognostic factor (Table 2).

To evaluate the independent prognostic value of the pretreatment LAR, two multivariate Cox regression models were constructed for each endpoint (Table 3). The baseline clinicopathological model (Model 1) incorporated age, ECOG performance status, MGMT promoter methylation status, multifocal disease, extent of resection, and Ki-67 labeling index. The augmented model (Model 2) additionally included the pretreatment LAR status using the cohort-derived cutoff value of 5.718.

For OS, the baseline clinicopathological model identified unmethylated MGMT promoter status as an independent prognostic factor (adjusted HR, 2.49; 95% CI, 1.43–4.32; *p* = 0.001), whereas ECOG performance status, multifocal disease, non-GTR, and Ki-67 index showed borderline associations. After including pretreatment LAR, an elevated LAR remained independently associated with shorter OS (adjusted HR, 4.51; 95% CI, 2.84–7.18; *p* < 0.001). In the augmented model, increasing age (adjusted HR, 1.02 per year; 95% CI, 1.00–1.04; *p* = 0.018) and non-GTR (adjusted HR, 1.59; 95% CI, 1.07–2.37; *p* = 0.023) also remained independently associated with worse OS, whereas the associations observed for MGMT promoter methylation status, ECOG performance status, multifocal disease, and Ki-67 index were attenuated after adjustment for LAR.

For PFS, the baseline clinicopathological model identified ECOG performance status, unmethylated MGMT promoter status, non-GTR, and a higher Ki-67 labeling index as independent prognostic variables. Following the inclusion of pretreatment LAR, an elevated LAR remained independently associated with shorter PFS (adjusted HR, 3.60; 95% CI, 2.33–5.56; *p* < 0.001). In the augmented model, non-GTR (adjusted HR, 1.57; 95% CI, 1.05–2.34; *p* = 0.027) and higher Ki-67 labeling index (adjusted HR, 1.01 per 1% increase; 95% CI, 1.004–1.021; *p* = 0.005) remained independently associated with shorter PFS, whereas the remaining clinicopathological variables were no longer statistically significant.

In a sensitivity analysis restricted to the 72 patients with known MGMT promoter methylation status, the association between high pretreatment LAR and adverse survival outcomes remained consistent with the primary analysis. High LAR remained independently associated with shorter OS (adjusted HR, 4.36; 95% CI, 2.26–8.40; *p* < 0.001) and PFS (adjusted HR, 3.35; 95% CI, 1.81–6.22; *p* < 0.001) after adjustment for age, ECOG performance status, MGMT promoter methylation status, multifocal disease, extent of resection, and Ki-67 labeling index.

Model performance analyses further demonstrated that the addition of pretreatment LAR improved prognostic performance beyond the baseline clinicopathological model for both survival endpoints (Table 4). For OS, the inclusion of LAR reduced the Akaike information criterion from 970.39 to 931.15, increased Harrell’s C-index from 0.699 to 0.760, and significantly improved the overall model fit (likelihood-ratio χ^2^ = 41.24; *p* < 0.001). Similar improvements were observed for PFS, with the AIC decreasing from 985.00 to 953.67, Harrell’s C-index increasing from 0.690 to 0.759, and a significant improvement in the model fit (likelihood-ratio χ^2^ = 33.33; *p* < 0.001).

### 3.5. Subgroup Analyses and Interaction Testing

The prognostic association between pretreatment LAR and survival outcomes was evaluated across clinicopathological subgroups using subgroup-specific Cox proportional hazard models (Figure 5). For OS, an elevated pretreatment LAR was associated with an increased risk of death across all evaluated subgroups (Figure 5A). Formal interaction testing demonstrated a significant interaction between LAR and age (*p* for interaction < 0.001), whereas no significant interactions were observed for sex (*p* = 0.869), ECOG performance status (*p* = 0.169), extent of resection (*p* = 0.144), tumor focality (*p* = 0.306), tumor size (*p* = 0.718), ATRX status (*p* = 0.524), or Ki-67 index (*p* = 0.420).

For PFS, an elevated pretreatment LAR was also associated with an increased risk of progression across all evaluated subgroups (Figure 5B). A significant interaction between LAR and age was observed (*p* < 0.001). No significant interactions were identified for sex (*p* = 0.465), ECOG performance status (*p* = 0.274), extent of resection (*p* = 0.384), tumor focality (*p* = 0.385), tumor size (*p* = 0.843), ATRX status (*p* = 0.411), or Ki-67 index (*p* = 0.675).

### 3.6. Landmark Survival Analyses

Landmark analyses were performed to assess whether the prognostic association between pretreatment LAR and survival outcomes persisted after excluding patients with early death or disease progression (Figure 6).

At the 3-month landmark, 123 patients were eligible for the OS analysis and 120 for the PFS analysis. High pretreatment LAR remained associated with shorter subsequent OS (HR, 3.73; 95% CI, 2.48–5.61; log-rank *p* < 0.001) and PFS (HR, 3.40; 95% CI, 2.26–5.12; log-rank *p* < 0.001) (Figure 6A).

At the 6-month landmark, 100 patients were included in the OS analysis and 92 in the PFS analysis. Elevated pretreatment LAR remained associated with an increased risk of death (HR, 2.76; 95% CI, 1.70–4.48; log-rank *p* < 0.001) and disease progression (HR, 2.59; 95% CI, 1.55–4.31; log-rank *p* < 0.001) among patients who remained event-free at the corresponding landmark time point (Figure 6B).

### 3.7. Association Between Pretreatment LAR and Treatment Response During Adjuvant Temozolomide

Among the 104 patients who received adjuvant temozolomide, the treatment response was evaluable in all patients. Outcomes were classified as disease control or PD according to a predefined response framework, with disease control encompassing CR and non-progressive residual disease.

Patients with PD had higher pretreatment LAR values than those with disease control (median, 5.57 [IQR, 4.98–6.86] vs. 4.33 [IQR, 3.94–5.37]; Mann–Whitney *p* < 0.001) (Figure 7A). Using the cohort-derived LAR cutoff of 5.718, PD occurred in 21/30 patients (70.0%) in the LAR-high group and 22/74 (29.7%) in the LAR-low group (Fisher exact *p* < 0.001) (Figure 7B).

Univariate logistic regression analyses revealed that unmethylated MGMT promoter status, multifocal disease, higher Ki-67 labeling index, and high pretreatment LAR were associated with PD. Age, ECOG performance status, extent of resection, and unknown MGMT promoter status were not statistically significant in the univariate analyses (Table 5).

The clinicopathological model yielded an AUC of 0.771, an AIC of 132.31, and a McFadden pseudo-R^2^ of 0.175. The addition of pretreatment LAR increased the AUC to 0.797, reduced the AIC to 128.24, and increased the McFadden pseudo-R^2^ to 0.218; the likelihood-ratio test confirmed improved model fit (χ^2^ = 6.07; *p* = 0.014) (Table 6). In the augmented model, a high pretreatment LAR remained independently associated with PD (adjusted OR, 3.72; 95% CI, 1.28–10.81; *p* = 0.016).

### 3.8. Internal Validation and Model Diagnostics

To further characterize the prognostic behavior of the pretreatment LAR, complementary internal validation and regression diagnostic analyses were performed (Figure 8). Bootstrap internal validation using 500 resamples demonstrated limited optimism in model discrimination, with apparent Harrell’s C-indices of 0.760 for OS and 0.759 for PFS, corresponding to optimism-corrected C-indices of 0.743 and 0.742, respectively (Figure 8A). Time-dependent evaluation of the adjusted effect of pretreatment LAR demonstrated attenuation of the hazard ratio during follow-up, with adjusted hazard ratios for LAR-high decreasing from 21.91 at 3 months to 6.55 at 6 months and 1.96 at 12 months for OS, whereas the corresponding estimates for PFS declined from 15.51 to 4.23 and 1.16, respectively (Figure 8B). Consistent with these findings, scaled Schoenfeld residual analyses demonstrated evidence of time-dependent variation in the effect of pretreatment LAR for both OS (LAR test, *p* = 0.040) and PFS (LAR test, *p* = 0.010) (Figure 8C,D). When modeled as a continuous variable using restricted cubic splines, pretreatment LAR demonstrated significant nonlinear associations with both OS and PFS (*p* for nonlinearity = 0.003 and 0.015, respectively), with increasing adjusted hazards across higher LAR values relative to the cohort-derived reference value of 5.718 (Figure 8E).

## 4. Discussion

Marked variations in the clinical course remain a defining challenge in IDH-wildtype glioblastoma, even among patients managed within broadly similar therapeutic pathways. In this real-world cohort, pretreatment LAR delineated a subgroup with a consistent pattern of early adverse clinical outcomes that extended beyond simple survival association. Patients with elevated LAR experienced substantially shorter overall and progression-free survival, and LAR retained independent prognostic significance after adjustment for established clinicopathological characteristics, while improving the performance of the baseline prognostic model. This unfavorable profile was also evident during treatment: PD was more frequent among patients with high LAR receiving adjuvant temozolomide, and the survival association persisted in landmark analyses that excluded the earliest deaths and progression events. Notably, the prognostic signal was concentrated toward the beginning of follow-up, with both discrimination and adjusted effect estimates diminishing over time. Together with the nonlinear risk gradient observed across continuous LAR values, these results support the interpretation that pretreatment LAR may identify patients who begin treatment with an adverse clinical trajectory already apparent at the systemic level. This interpretation remains prognostic rather than mechanistic or treatment-predictive but provides a clinically relevant framework for considering LAR as a complement to the conventional baseline prognostic assessment.

The continuous analyses also place the dichotomous LAR classification in context. Restricted cubic spline modeling demonstrated significant nonlinear associations with both OS and PFS, indicating that prognostic information is distributed across the LAR continuum rather than arising exclusively at a single threshold. Accordingly, the cohort-derived cut-off of 5.718 should not be interpreted as a biological boundary or as evidence of an abrupt change in risk. Its principal value in the present study was pragmatic and descriptive, providing an interpretable framework for Kaplan–Meier visualization and complementary subgroup, landmark, and treatment-response analyses. The continuous and categorical analyses should therefore be regarded as complementary: the former more faithfully characterizes the shape of the prognostic association, whereas the latter provides an exploratory framework for clinically interpretable risk stratification.

The biological rationale for LAR lies in its integration of circulating signals related to both disease-associated stress and host physiological reserve. LDH is influenced by cellular turnover, tissue injury, hypoxic stress, and altered energy metabolism [14,15], whereas circulating albumin is shaped by inflammatory activity, hepatic synthetic capacity, vascular permeability, nutritional status, and the systemic response to illness [16]. An elevated LAR may therefore arise when greater metabolic or tissue stress is accompanied by diminished host reserve, providing a composite representation of two biologically distinct but clinically convergent processes. This framework is particularly relevant to glioblastoma, where profound metabolic adaptation, regional hypoxia, a prominent myeloid compartment, and systemic inflammatory responses coexist within a highly heterogeneous disease state [27]. Importantly, circulating LAR cannot resolve these individual processes or establish their biological origin. Rather, LAR may be viewed as a nonspecific systemic composite reflecting aspects of the physiological context accompanying an adverse clinical state, without serving as a direct measure of tumor metabolism, hypoxia, lactate production, or immune activity. This interpretation is consistent with the broader prognostic literature on inflammation- and nutrition-related indices in glioma [28], while extending that framework by incorporating LDH as a component associated with tissue and metabolic stress. Defining the biological basis of LAR will require studies that integrate circulating measurements with tumor-level assessments of metabolism, hypoxia, lactate biology, and immune-cell states.

The LDH component provides a biologically plausible connection to the metabolic landscape of glioblastoma, although this relationship requires careful distinction between circulating and tumor-level biology. Glioblastoma cells retain substantial metabolic plasticity, with enhanced glycolytic flux and lactate production supporting adaptation to hypoxic and nutrient-constrained regions of the tumor microenvironment [29]. Beyond its role as a metabolic end product, lactate can participate in intercellular signaling and immune regulation, thereby linking altered tumor metabolism to the composition and functional state of the surrounding microenvironment [30,31]. Recent experimental evidence has further implicated LDHA-dependent metabolism in reciprocal interactions between glioblastoma cells and tumor-associated macrophages, including chemokine-mediated macrophage recruitment and macrophage-derived signals that reinforce tumor-cell glycolysis, proliferation, and survival []. These observations provide a biological context for investigating an LDH-containing circulating biomarker in glioblastoma but do not establish equivalence between systemic LDH measurements and intratumoral metabolic processes. Serum total LDH cannot be assumed to reflect tumor LDHA expression or activity, glycolytic flux, intratumoral lactate production, or tumor hypoxia, and the present findings therefore cannot establish a causal relationship between these mechanisms and circulating LAR. Rather, elevated pretreatment LAR should be interpreted as a nonspecific systemic correlate of an adverse clinical state whose underlying biological determinants remain to be established. Integrating circulating LAR with tumor metabolomics, metabolic imaging, hypoxia-related markers, and spatial or single-cell immune profiling will be necessary to determine whether this systemic signal tracks specific features of glioblastoma biology.

Albumin adds a complementary host-related dimension to LAR. Although traditionally regarded as a marker of nutritional status, circulating albumin is also shaped by systemic inflammation, hepatic synthetic function, vascular permeability, and the broader physiological response to illness [17,32]. In patients with glioblastoma, these influences may intersect with disease-related catabolism, corticosteroid exposure, neurological disability, reduced nutritional intake, and the cumulative physiological burden of treatment [33], making albumin less a specific measure of nutrition than an integrated marker of host condition. This distinction is relevant when LAR is considered alongside inflammatory indices derived predominantly from circulating immune-cell populations. NLR, SII, SIRI, PIV, and related measures have all been investigated as prognostic markers in glioblastoma, although the magnitude and consistency of their associations with outcome vary across studies [9,10,11,23]. In our cohort, several of these indices were associated with adverse outcomes, but head-to-head analyses provided little evidence that LAR was uniformly more discriminatory: after correction for multiple comparisons, only the comparison with MAR remained significant for 12-month OS. LAR should therefore not be regarded as a replacement for established inflammatory indices. Its potential value may instead lie in integrating an LDH-related signal of tissue and metabolic stress with an albumin-related dimension of host condition. Whether this combination provides reproducible prognostic information beyond existing systemic biomarkers will require validation in independent cohorts using harmonized, head-to-head analytical frameworks.

A notable feature of the LAR signal was its temporal concentration early in the disease course. Both prognostic discrimination and the adjusted excess hazard associated with elevated LAR were greatest soon after diagnosis and attenuated with longer follow-up, suggesting that the information captured by a pretreatment measurement may be most relevant to the early clinical trajectory. This pattern was not attributable solely to events occurring immediately after diagnosis, as the adverse association persisted in landmark analyses restricted to patients who had survived, or remained alive and progression-free, beyond the first 3 and 6 months. The treatment-response findings provided a complementary clinical correlate of the same phenotype: among patients receiving adjuvant temozolomide, elevated LAR was associated with a substantially greater likelihood of PD and remained independently associated with progression after adjustment for clinicopathological factors. Taken together, the temporal survival pattern and response findings support a model in which elevated pretreatment LAR identifies patients entering treatment with an adverse systemic and clinical state whose consequences become apparent relatively early in the disease course. These observations should not, however, be interpreted as evidence of temozolomide-specific resistance. Without an untreated or alternative-treatment comparator, an association with progression during temozolomide cannot distinguish treatment-specific resistance from an underlying adverse disease course. The response analysis therefore provides convergent evidence of an early adverse prognostic profile rather than evidence that LAR is predictive of temozolomide benefit.

The practical relevance of the LAR derives chiefly from its availability. LDH and albumin are inexpensive routine measurements that can be obtained before treatment without additional tissue acquisition, specialized imaging, or complex molecular assays. In our cohort, incorporating LAR into the clinicopathological model improved both model fit and discrimination for overall and progression-free survival, suggesting that systemic information present at diagnosis may add to conventional prognostic assessment. However, these findings do not establish LAR as a treatment-predictive biomarker and therefore do not support therapeutic escalation, de-escalation, or selection of alternative treatment strategies according to LAR status. A more realistic translational role would be as one component of multidimensional baseline risk characterization, integrated with performance status, surgical factors, MGMT promoter methylation, imaging, and emerging molecular markers. If externally validated, LAR could subsequently be incorporated into prospective biomarker-stratified studies to determine whether patients with different baseline LAR profiles derive differential benefit from specific therapeutic strategies. External validation is also required to establish whether its incremental prognostic information is reproducible across contemporary glioblastoma populations and whether longitudinal LAR measurements provide information beyond a single pretreatment assessment. Whether this prognostic signal extends to other primary or secondary intracranial malignancies remains unknown. Given the biological and clinical heterogeneity across CNS tumor entities, the prognostic value and clinically relevant range of LAR should be evaluated independently within each disease context rather than extrapolated directly from IDH-wildtype glioblastoma.

Several features strengthen the interpretation of these findings, particularly the convergence of complementary analytical approaches on the same clinical signal; however, important limitations define the scope of the inference. The retrospective, single-center design carries an inherent risk of selection bias, residual confounding, and center-specific treatment and follow-up patterns, which may limit its generalizability. The LAR threshold was derived and evaluated within the same cohort; although bootstrap analyses suggested internal stability and limited optimism, internal validation cannot replace independent external validation, and the cut-off of 5.718 should therefore be considered exploratory and cohort-derived rather than clinically established. MGMT promoter methylation status was unavailable in a substantial proportion of patients because of historical variations in molecular testing. However, in a sensitivity analysis restricted to patients with known MGMT status, the association between elevated LAR and both OS and PFS remained consistent with the primary analysis. Nevertheless, the substantial MGMT missingness and reduced sample size of this sensitivity cohort warrant cautious interpretation and do not eliminate the possibility of residual molecular confounding. The treatment-response analysis was confined to patients who received adjuvant temozolomide and had an evaluable response, introducing potential selection and survivorship effects and preventing treatment-specific predictive inference. Moreover, LDH and albumin levels are nonspecific circulating measures that may be influenced by inflammatory, hepatic, nutritional, metabolic, and other systemic conditions unrelated to the tumor. Finally, the absence of paired tissue-based, metabolic, hypoxia-related, immune, and longitudinal circulating measurements precludes direct mechanistic attribution and prevents circulating LAR from being interpreted as a surrogate for specific intratumoral biological processes.

## 5. Conclusions

Pretreatment LAR identified an adverse prognostic profile in IDH-wildtype glioblastoma, with its prognostic signal most pronounced early in the disease course. Elevated LAR was associated with poorer survival and a greater likelihood of progression during adjuvant temozolomide while providing incremental prognostic information beyond conventional clinicopathological characteristics. These findings do not currently support LAR-guided treatment selection; rather, LAR may provide an accessible component of baseline risk characterization that complements established prognostic assessment and warrants evaluation in prospective biomarker-stratified studies. Independent validation in contemporary, molecularly characterized cohorts is required to establish the reproducibility and clinical relevance of this signal.

## Figures and Tables

**Figure 1:**
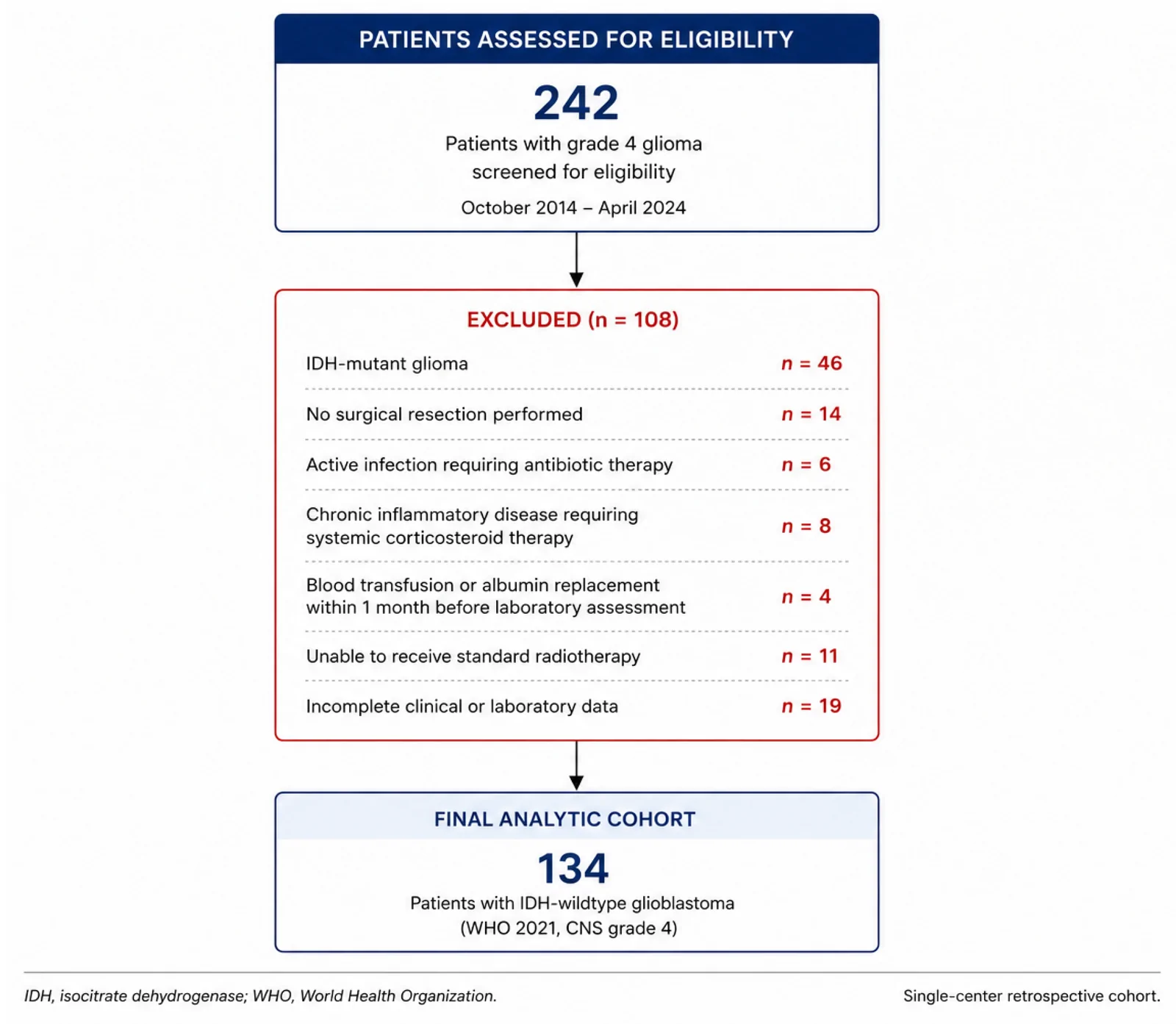
Patient selection and derivation of the final analytic cohort.

**Figure 4:**
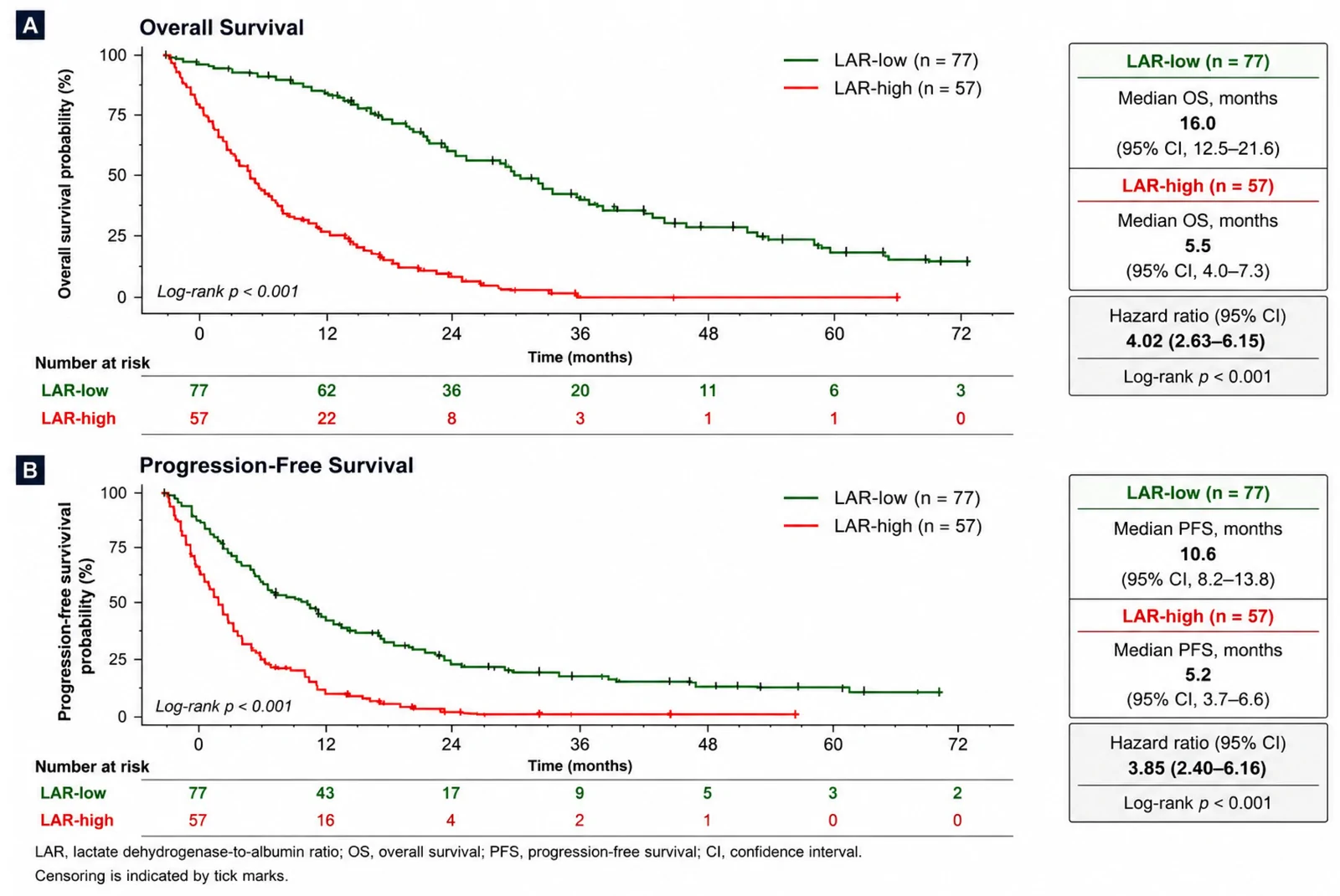
Kaplan–Meier estimates of overall survival (**A**) and progression-free survival (**B**) according to pretreatment lactate dehydrogenase-to-albumin ratio (LAR) status using the cohort-derived cut-off value of 5.718. Hazard ratios were estimated using univariable Cox proportional hazards models. Tick marks indicate censored observations, and numbers below the plots represent patients at risk at each time point. Abbreviations: CI, confidence interval; HR, hazard ratio; LAR, lactate dehydrogenase-to-albumin ratio; OS, overall survival; PFS, progression-free survival.

**Figure 5:**
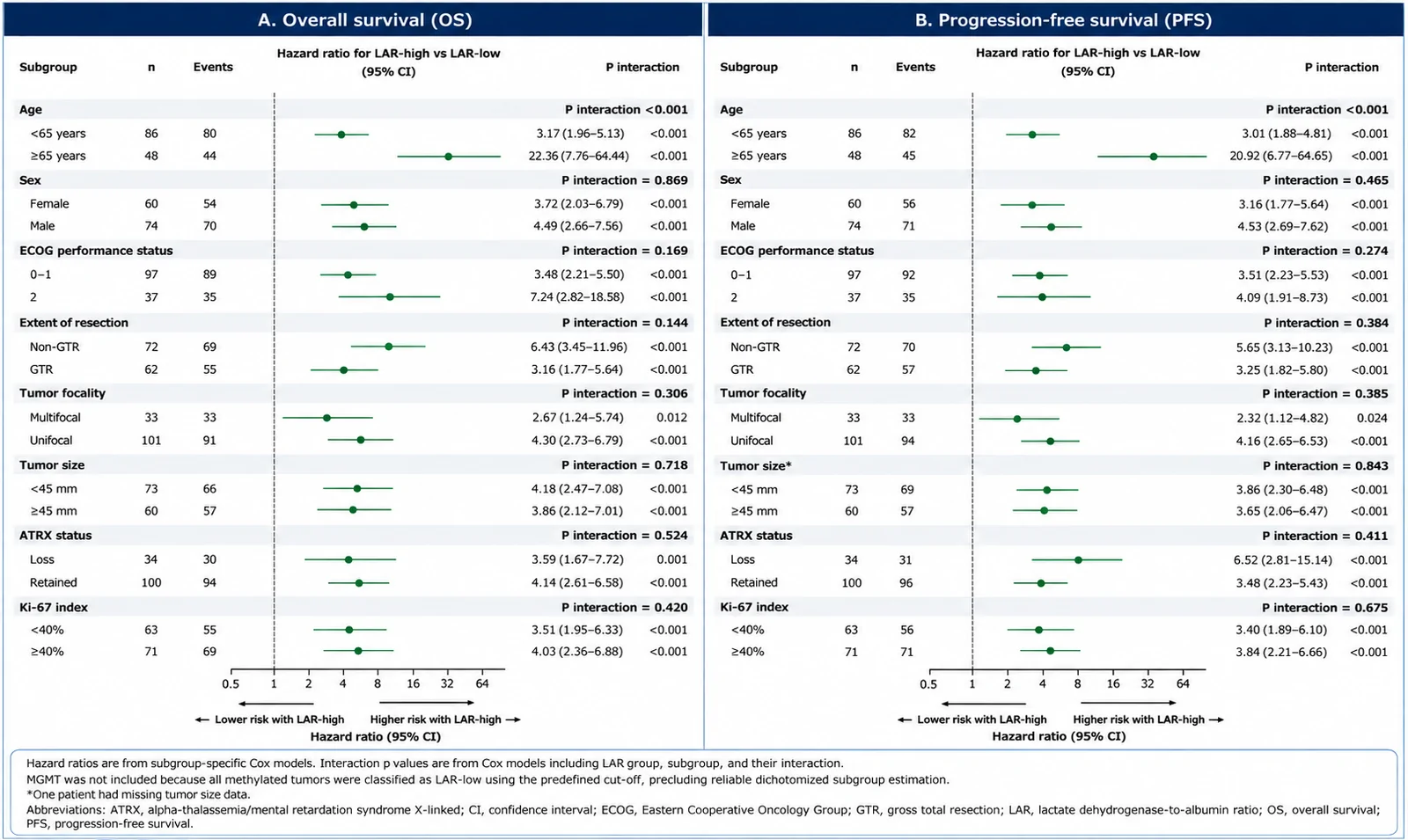
Subgroup analyses of the association between pretreatment lactate dehydrogenase-to-albumin ratio and survival outcomes. Forest plots showing subgroup-specific hazard ratios and 95% confidence intervals for overall survival (**A**) and progression-free survival (**B**), comparing patients with high versus low pretreatment LAR. Interaction *p* values were derived from Cox proportional hazards models, including the LAR group, subgroup membership, and the corresponding interaction term. The vertical dashed line indicates a hazard ratio of 1.0.

**Figure 6:**
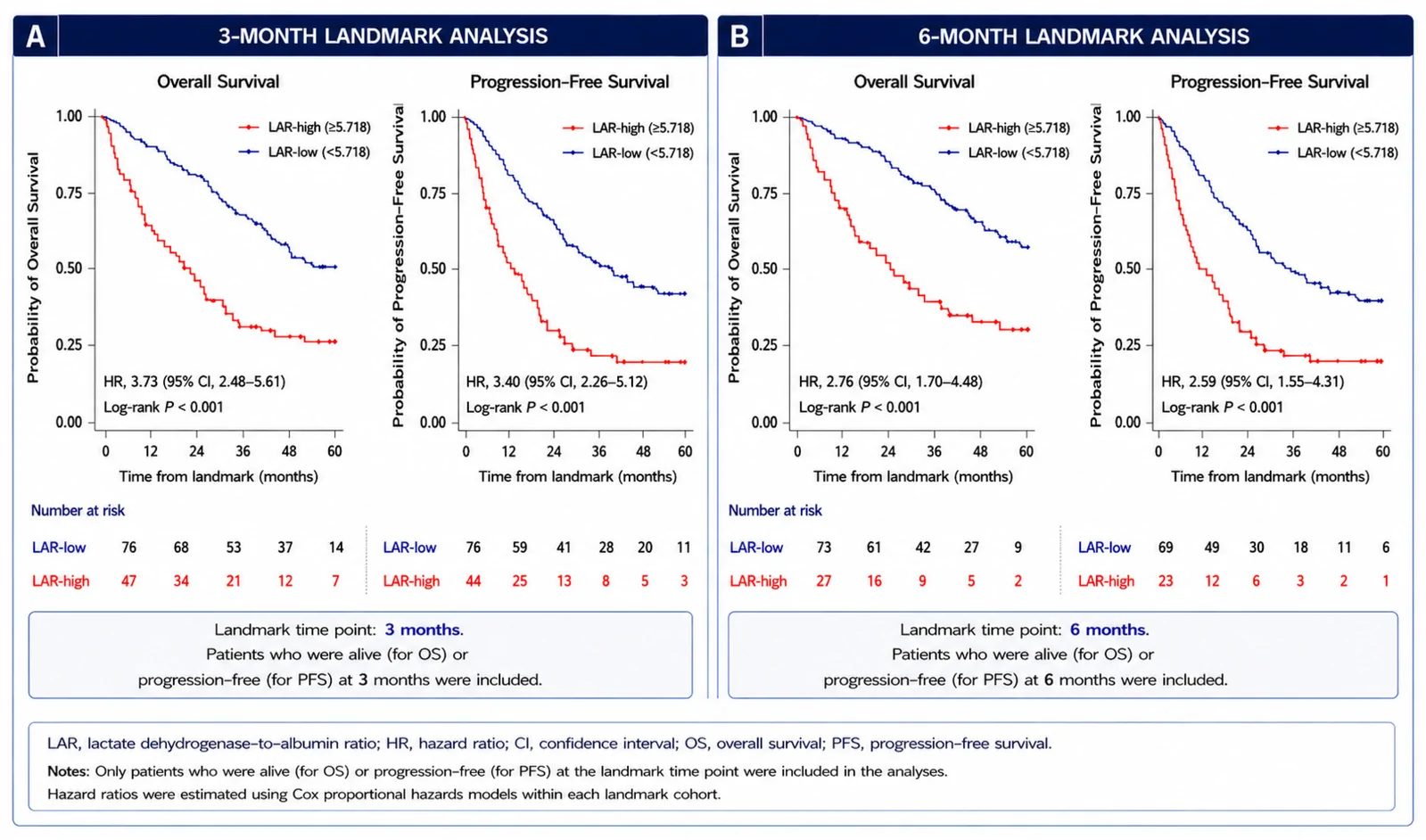
Landmark Kaplan–Meier analyses according to pretreatment lactate dehydrogenase-to-albumin ratio. Kaplan–Meier curves for overall survival (OS) and progression-free survival (PFS) after 3-month (**A**) and 6-month (**B**) landmark analyses. Only patients who remained alive (for OS) or progression-free (for PFS) at the corresponding landmark time points were included. Hazard ratios (HRs) and 95% confidence intervals (CIs) were estimated using Cox proportional hazards models for each landmark cohort. The number of patients at risk is shown below each panel.

**Figure 7:**
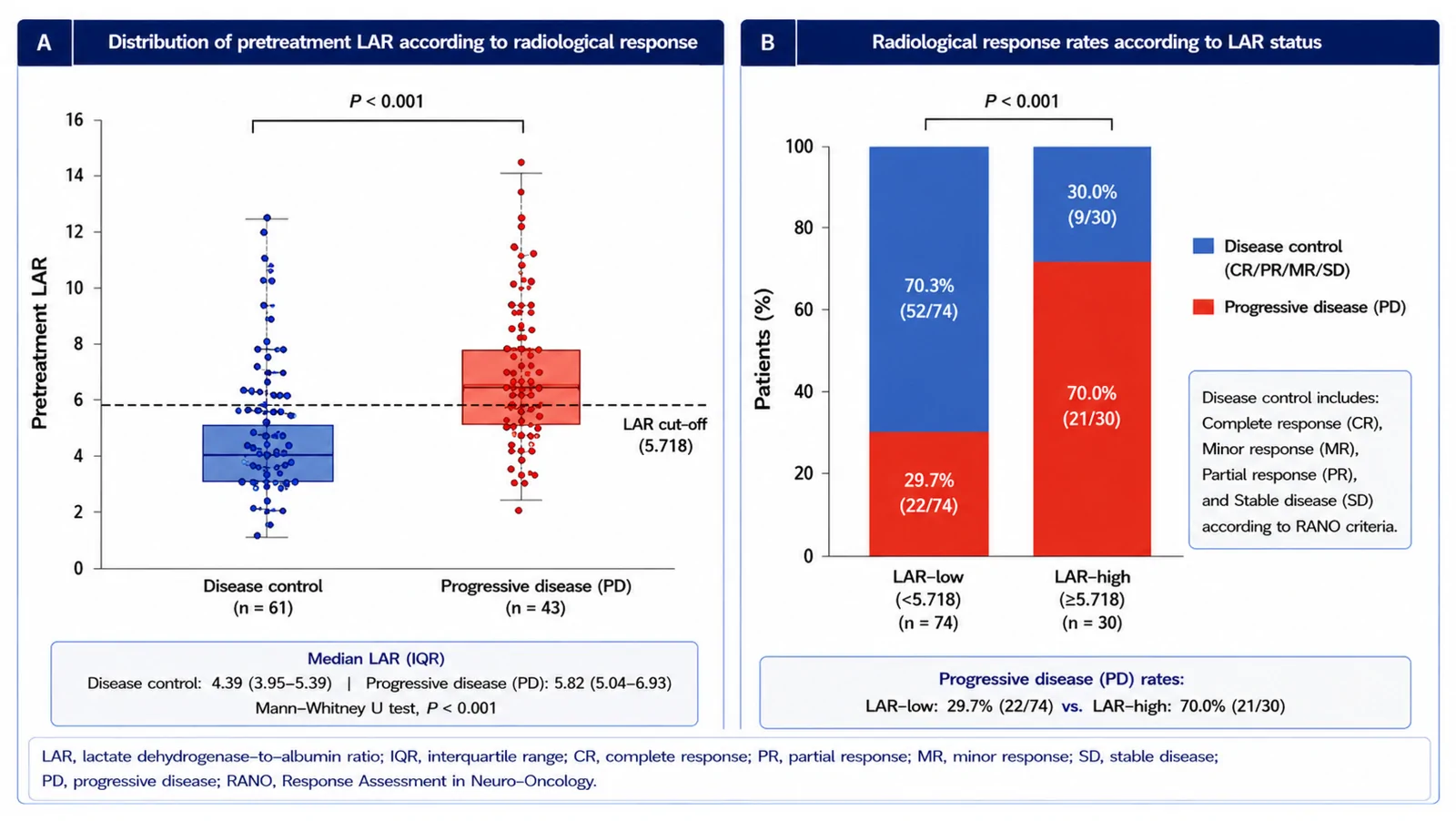
Pretreatment lactate dehydrogenase-to-albumin ratio and treatment response during adjuvant temozolomide. Panel (**A**) shows pretreatment lactate dehydrogenase-to-albumin ratio (LAR) according to treatment-response category. Each circle represents an individual patient’s pretreatment LAR value; blue circles indicate patients with disease control, whereas red circles indicate patients with progressive disease. The horizontal dashed line indicates the cohort-derived LAR cut-off value of 5.718. Panel (**B**) shows the proportions of patients with disease control and progressive disease according to pretreatment LAR status. Disease control encompassed a complete response and non-progressive residual disease. Statistical comparisons were performed using the Mann–Whitney U test (Panel (**A**)) and Fisher’s exact test (Panel (**B**)).

**Figure 8:**
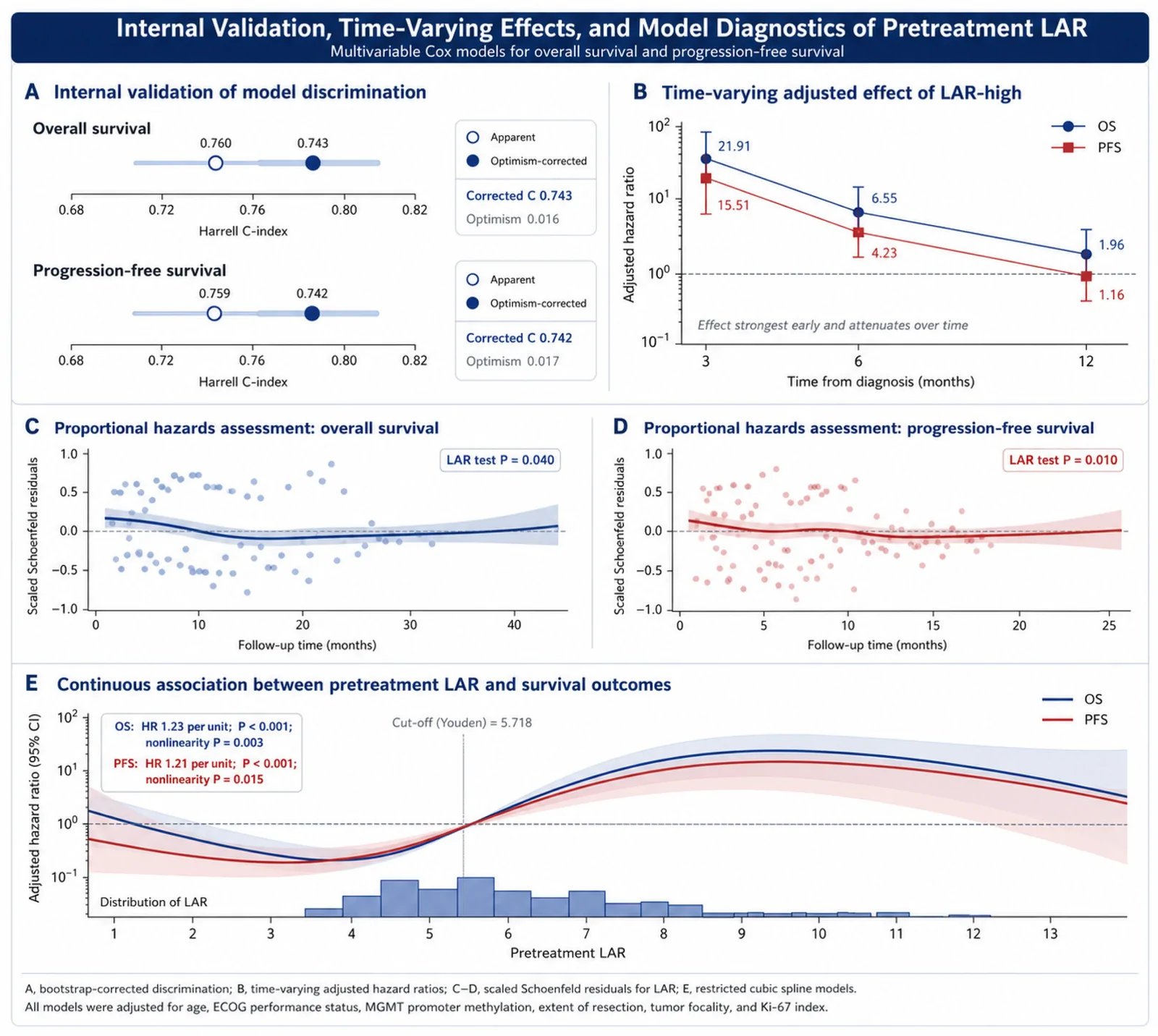
Internal validation and time-dependent prognostic behavior of pretreatment LAR. (**A**) Internal bootstrap validation of the multivariable Cox models showing apparent and optimism-corrected Harrell’s C-indices for overall survival (OS) and progression-free survival (PFS). (**B**) Time-varying adjusted hazard ratios for patients with high pretreatment LAR, illustrating changes in effect estimates over follow-up. The horizontal dashed line indicates the null reference (hazard ratio = 1). (**C**,**D**) Scaled Schoenfeld residual plots assessing the proportional hazards assumption for pretreatment LAR in the OS and PFS models, respectively. The light blue and light red circles represent individual scaled Schoenfeld residuals, the corresponding solid curves show smoothed trends over follow-up, and the shaded bands indicate uncertainty around these fitted trends. The horizontal dashed lines indicate zero scaled Schoenfeld residuals. (**E**) Restricted cubic spline analyses depicting the continuous association between pretreatment LAR and adjusted hazard ratios for OS and PFS. Solid lines represent adjusted hazard ratios, shaded bands indicate 95% confidence intervals, and the histogram illustrates the distribution of pretreatment LAR. The horizontal dashed line indicates the null reference (hazard ratio = 1), while the vertical dotted line denotes the cohort-derived cut-off value (LAR = 5.718). Footnote: Bootstrap internal validation was performed using 500 resamples. All multivariable Cox models were adjusted for age, ECOG performance status, MGMT promoter methylation status, multifocal disease, extent of resection, and Ki-67 proliferation index. The hazard ratios from the restricted cubic spline analyses are presented relative to the cohort-derived reference value (LAR = 5.718).

**Table 1:** Baseline Characteristics According to Pretreatment Lactate Dehydrogenase-to-Albumin Ratio Status (*n* = 134).

Variable	Category	Overall Cohort (*n* = 134)	LAR-Low (*n* = 77)	LAR-High (*n* = 57)	*p* Value
Age, years		60.0 (52.2–67.0)	60.0 (53.0–67.0)	61.0 (51.0–66.0)	0.787
Sex	Male	74 (55.2)	40 (51.9)	34 (59.6)	0.477
	Female	60 (44.8)	37 (48.1)	23 (40.4)	
Comorbidity	Yes	61 (45.5)	34 (44.2)	27 (47.4)	0.846
	No	73 (54.5)	43 (55.8)	30 (52.6)	
Smoking	Yes	51 (38.1)	26 (33.8)	25 (43.9)	0.313
	No	83 (61.9)	51 (66.2)	32 (56.1)	
ECOG performance status	0–1	97 (72.4)	60 (77.9)	37 (64.9)	0.142
	2	37 (27.6)	17 (22.1)	20 (35.1)	
Body mass index, kg/m^2^		26.8 (23.1–29.5)	28.1 (25.4–30.5)	24.2 (21.5–27.2)	<0.001
Neurological deficit	Yes	28 (20.9)	14 (18.2)	14 (24.6)	0.495
	No	106 (79.1)	63 (81.8)	43 (75.4)	
Epileptic seizure	Yes	35 (26.1)	24 (31.2)	11 (19.3)	0.178
	No	99 (73.9)	53 (68.8)	46 (80.7)	
Tumor lateralization	Left	71 (53.0)	35 (45.5)	36 (63.2)	0.064
	Right	63 (47.0)	42 (54.5)	21 (36.8)	
Tumor focality	Unifocal	101 (75.4)	62 (80.5)	39 (68.4)	0.160
	Multifocal	33 (24.6)	15 (19.5)	18 (31.6)	
Tumor location	Temporal	40 (29.9)	24 (31.2)	16 (28.1)	0.452
	Parietal	40 (29.9)	24 (31.2)	16 (28.1)	
	Frontal	38 (28.4)	18 (23.4)	20 (35.1)	
	Other	16 (11.9)	11 (14.3)	5 (8.8)	
Tumor size, mm		44.0 (35.0–53.0)	44.0 (32.5–52.2)	43.0 (36.0–55.0)	0.547
Extent of resection	Non-GTR	72 (53.7)	38 (49.4)	34 (59.6)	0.314
	GTR	62 (46.3)	39 (50.6)	23 (40.4)	
MGMT promoter methylation	Unmethylated	54 (40.3)	22 (28.6)	32 (56.1)	<0.001
	Methylated	18 (13.4)	13 (16.9)	5 (8.8)	
	Unknown	62 (46.3)	42 (54.5)	20 (35.1)	
Ki-67, %		40.0 (20.0–60.0)	30.0 (20.0–50.0)	40.0 (25.0–60.0)	0.066
Adjuvant chemotherapy	Yes	104 (77.6)	65 (84.4)	39 (68.4)	<0.001
	No	30 (22.4)	12 (15.6)	18 (31.6)	
Total duration of adjuvant chemotherapy, months		6.0 (6.0–8.0)	6.0 (6.0–12.0)	6.0 (4.0–6.0)	<0.001
Surgery performed at progression	Yes	39 (41.1)	30 (46.2)	9 (30.0)	0.206
	No	56 (58.9)	35 (53.8)	21 (70.0)	
Systemic chemotherapy at progression	Yes	47 (49.5)	37 (56.9)	10 (33.3)	0.055
	No	48 (50.5)	28 (43.1)	20 (66.7)	
NLR		2.92 (2.12–4.37)	2.41 (1.65–3.37)	3.72 (2.82–5.10)	<0.001
SII		9.32 (5.98–15.12)	6.87 (4.82–10.57)	13.38 (8.87–20.19)	<0.001
SIRI		18.77 (12.07–28.94)	17.18 (9.90–25.67)	22.94 (16.37–32.55)	0.002
PIV		57.84 (32.08–101.24)	43.77 (25.66–83.05)	76.18 (51.57–133.31)	<0.001
CAR		8.24 (4.54–19.03)	5.41 (3.59–9.38)	17.11 (8.95–27.65)	<0.001
MAR		15.79 (11.65–21.85)	15.61 (11.74–21.62)	16.92 (11.52–22.82)	0.521

Data are presented as median (IQR) for continuous variables and as *n* (%) for categorical variables. *p*-values were calculated using the Mann–Whitney U test for continuous variables and χ^2^ or Fisher’s exact test for categorical variables, as appropriate. Percentages were calculated using the available non-missing data for each variable. LAR, lactate dehydrogenase-to-albumin ratio; GTR, gross total resection; MGMT, O-6-methylguanine-DNA methyltransferase; NLR, neutrophil-to-lymphocyte ratio; SII, systemic immune-inflammation index; SIRI, systemic inflammation response index; PIV, pan-immune-inflammation value; CAR, C-reactive protein-to-albumin ratio; MAR, monocyte-to-albumin ratio. Note: Tumor lateralization was coded as left/right according to the dataset coding dictionary. Tumor locations labelled as occipital or cerebellar were grouped as other.

**Table 2:** Univariable Cox Regression Analyses for Overall Survival and Progression-Free Survival.

Variable	Overall Survival		Progression-Free Survival	
	HR (95% CI)	*p*	HR (95% CI)	*p*
Age, per year	1.01 (0.99–1.02)	0.370	1.01 (0.99–1.02)	0.436
Female sex	1.00 (0.70–1.43)	0.992	1.05 (0.74–1.49)	0.785
ECOG 2 vs. 0–1	1.61 (1.09–2.40)	0.017	1.70 (1.14–2.53)	0.009
Comorbidity	0.98 (0.69–1.40)	0.908	1.01 (0.71–1.44)	0.955
Neurological deficit	1.23 (0.80–1.89)	0.349	1.19 (0.77–1.84)	0.431
Epileptic seizure	0.86 (0.57–1.29)	0.460	0.94 (0.63–1.40)	0.754
Tumor size, per mm	1.01 (0.99–1.02)	0.379	1.01 (0.99–1.02)	0.360
Multifocal disease	2.12 (1.41–3.20)	<0.001	2.02 (1.35–3.04)	<0.001
Tumor location: Frontal (reference)	Reference	0.922	Reference	0.888
Tumor location: Temporal vs. Frontal	1.15 (0.72–1.83)	0.557	1.09 (0.69–1.72)	0.722
Tumor location: Parietal vs. Frontal	1.16 (0.73–1.83)	0.533	1.12 (0.71–1.76)	0.627
Tumor location: Other vs. Frontal	1.11 (0.59–2.11)	0.746	1.28 (0.69–2.38)	0.441
Non-GTR vs. GTR	1.75 (1.22–2.52)	0.003	1.82 (1.27–2.61)	0.001
MGMT unmethylated vs. methylated	2.59 (1.48–4.51)	<0.001	2.83 (1.62–4.93)	<0.001
Ki-67, per 1%	1.01 (1.00–1.02)	0.019	1.01 (1.01–1.02)	<0.001
Adjuvant TMZ duration, per month	0.80 (0.74–0.87)	<0.001	0.76 (0.70–0.83)	<0.001
Progressive disease vs. Disease control	2.93 (1.94–4.44)	<0.001	3.82 (2.50–5.84)	<0.001
LAR-high (≥5.718)	4.02 (2.73–5.93)	<0.001	3.85 (2.63–5.66)	<0.001
NLR-high (≥2.615)	2.06 (1.43–2.97)	<0.001	2.14 (1.49–3.08)	<0.001
SII-high (≥10.188)	3.44 (2.36–4.99)	<0.001	4.25 (2.85–6.36)	<0.001
SIRI-high (≥15.142)	2.31 (1.57–3.39)	<0.001	2.02 (1.40–2.91)	<0.001
PIV-high (≥51.572)	2.68 (1.84–3.90)	<0.001	2.63 (1.82–3.80)	<0.001
CAR-high (≥7.353)	2.59 (1.79–3.73)	<0.001	2.73 (1.89–3.93)	<0.001
MAR-high (≥14.474)	1.74 (1.21–2.50)	0.003	1.74 (1.22–2.50)	0.003

Abbreviations: CAR, C-reactive protein-to-albumin ratio; CI, confidence interval; ECOG, Eastern Cooperative Oncology Group; GTR, gross total resection; HR, hazard ratio; LAR, lactate dehydrogenase-to-albumin ratio; MAR, monocyte-to-albumin ratio; MGMT, O6-methylguanine-DNA methyltransferase; NLR, neutrophil-to-lymphocyte ratio; PIV, pan-immune-inflammation value; SII, systemic immune-inflammation index; SIRI, systemic inflammation response index; TMZ, temozolomide. Note: Hazard ratios were estimated using separate univariable Cox proportional hazards models. For the MGMT promoter methylation status, the analysis was restricted to methylated and unmethylated tumors; unknown MGMT status was excluded. Tumor location was entered as a categorical variable, with frontal location as the reference category. Inflammatory biomarkers were dichotomized using 12-month overall survival ROC-derived cut-offs; LAR cut-off = 5.718.

**Table 3:** Multivariable Cox Regression Models for Overall Survival and Progression-Free Survival.

Variable	Overall Survival				Progression-Free Survival			
	Model 1 Adj HR (95% CI)	*p*	Model 2 Adj HR (95% CI)	*p*	Model 1 Adj HR (95% CI)	*p*	Model 2 Adj HR (95% CI)	*p*
Age, per year	1.005 (0.989–1.021)	0.563	1.021 (1.003–1.038)	0.018	1.006 (0.990–1.022)	0.490	1.015 (0.998–1.032)	0.080
ECOG 2 vs. 0–1	1.502 (0.994–2.271)	0.054	1.428 (0.936–2.179)	0.098	1.616 (1.064–2.454)	0.024	1.504 (0.981–2.305)	0.061
MGMT unmethylated vs. methylated	2.485 (1.430–4.319)	0.001	1.531 (0.847–2.767)	0.159	2.514 (1.430–4.420)	0.001	1.597 (0.868–2.937)	0.132
MGMT unknown vs. methylated	1.646 (0.949–2.855)	0.076	1.157 (0.650–2.060)	0.620	1.609 (0.918–2.822)	0.097	1.198 (0.668–2.147)	0.544
Multifocal disease	1.521 (0.984–2.351)	0.059	1.320 (0.850–2.049)	0.216	1.349 (0.864–2.105)	0.188	1.277 (0.817–1.997)	0.283
Non-GTR vs. GTR	1.432 (0.966–2.121)	0.074	1.589 (1.067–2.365)	0.023	1.505 (1.015–2.231)	0.042	1.571 (1.054–2.342)	0.027
Ki-67, per 1%	1.009 (1.000–1.018)	0.051	1.008 (0.999–1.017)	0.081	1.013 (1.005–1.022)	0.002	1.012 (1.004–1.021)	0.005
LAR-high (≥5.718)	—	—	4.514 (2.839–7.176)	<0.001	—	—	3.603 (2.334–5.561)	<0.001

Footnote: Model 1 included baseline clinicopathological variables (age, ECOG performance status, MGMT promoter methylation status, multifocal disease, extent of resection, and Ki-67 index). Model 2 included all variables from Model 1 with the addition of pretreatment lactate dehydrogenase-to-albumin ratio (LAR) status (high vs. low; cut-off, 5.718). Continuous variables were modeled per one-unit increase, unless otherwise specified. Hazard ratios (HRs) were estimated using Cox proportional hazards regression and are presented with corresponding 95% confidence intervals (CIs). Adjuvant temozolomide duration was intentionally excluded from the primary multivariable models because it represented a post-baseline treatment variable rather than a baseline prognostic characteristic. Abbreviations: CI, confidence interval; ECOG, Eastern Cooperative Oncology Group; GTR, gross total resection; HR, hazard ratio; Ki-67, Ki-67 labeling index; LAR, lactate dehydrogenase-to-albumin ratio; MGMT, O6-methylguanine-DNA methyltransferase.

**Table 4:** Incremental prognostic performance after addition of pretreatment LAR.

Endpoint	Model	*n*/Events	Model Performance		Increment After Adding LAR		
			AIC	Harrell C	ΔAIC	ΔHarrell C	Likelihood-Ratio Test
OS	Baseline clinicopathological model	134/124	970.39	0.699	—	—	—
OS	Baseline clinicopathological model + LAR	134/124	931.15	0.760	−39.24	+0.061	χ^2^ = 41.24; *p* < 0.001
PFS	Baseline clinicopathological model	134/127	985.00	0.690	—	—	—
PFS	Baseline clinicopathological model + LAR	134/127	953.67	0.759	−31.33	+0.069	χ^2^ = 33.33; *p* < 0.001

Model definitions: The baseline clinicopathological model included age, ECOG performance status, MGMT promoter methylation status, multifocal disease, extent of resection, and Ki-67 index. The augmented model additionally included a high pretreatment LAR (≥5.718). Negative ΔAIC and positive ΔHarrell C values indicate improved model fit and discrimination. Likelihood ratio tests compare the augmented model with the corresponding baseline model. Abbreviations: AIC, Akaike information criterion; LAR, lactate dehydrogenase-to-albumin ratio; OS, overall survival; PFS, progression-free survival.

**Table 5:** Logistic Regression Models for Progressive Disease During Adjuvant Temozolomide.

Variable	Univariable		Clinicopathological Model		Clinicopathological Model + LAR	
	OR (95% CI)	*p*	OR (95% CI)	*p*	OR (95% CI)	*p*
Age, per year	0.99 (0.95–1.02)	0.505	0.99 (0.95–1.03)	0.550	1.00 (0.96–1.05)	0.962
ECOG 2 vs. 0–1	2.50 (0.96–6.55)	0.061	3.16 (1.06–9.40)	0.039	3.19 (1.03–9.86)	0.044
MGMT unmethylated vs. methylated	7.50 (1.83–30.78)	0.005	7.35 (1.52–35.53)	0.013	4.54 (0.89–23.19)	0.069
MGMT unknown vs. methylated	2.97 (0.76–11.61)	0.118	2.89 (0.65–12.98)	0.165	2.00 (0.43–9.39)	0.380
Multifocal disease	2.73 (1.09–6.88)	0.033	2.02 (0.69–5.89)	0.199	1.71 (0.56–5.21)	0.347
Non-GTR vs. GTR	1.16 (0.53–2.52)	0.716	0.84 (0.33–2.15)	0.722	0.82 (0.31–2.17)	0.695
Ki-67, per 1%	1.03 (1.01–1.05)	0.003	1.03 (1.00–1.05)	0.017	1.03 (1.00–1.05)	0.023
LAR-high (≥5.718)	5.52 (2.18–13.93)	<0.001	—	—	3.72 (1.28–10.81)	0.016

Notes: The outcome was progressive disease versus disease control during adjuvant temozolomide. Disease control encompassed a complete response and non-progressive residual disease. The clinicopathological model included age, ECOG performance status, MGMT promoter methylation status, multifocal disease, extent of resection, and Ki-67 labeling index. The augmented model additionally included pretreatment LAR status (high vs. low; cut-off, 5.718). Analyses included all 104 patients who received adjuvant temozolomide and had evaluable treatment responses. Abbreviations: CI, confidence interval; ECOG, Eastern Cooperative Oncology Group; GTR, gross total resection; LAR, lactate dehydrogenase-to-albumin ratio; MGMT, O6-methylguanine-DNA methyltransferase; OR, odds ratio.

**Table 6:** Incremental Model Performance After Addition of Pretreatment LAR for Progressive Disease.

Model	*n*/Events	Model Performance			Increment After Adding LAR		
		AIC	AUC	McFadden R^2^	ΔAIC	ΔAUC/ΔR^2^	Likelihood-Ratio Test
Clinicopathological model	104/43	132.31	0.771	0.175	—	—	—
Clinicopathological model + LAR	104/43	128.24	0.797	0.218	−4.07	+0.026/+0.043	χ^2^ = 6.07; *p* = 0.014

Notes: The clinicopathological model included age, ECOG performance status, MGMT promoter methylation status, multifocal disease, extent of resection, and Ki-67 labeling index. The augmented model included a high pretreatment LAR (≥5.718). Negative ΔAIC and positive ΔAUC and ΔMcFadden R^2^ values indicated improved model fit and discrimination. The likelihood-ratio test compared the augmented model with the clinicopathological model. Abbreviations: AIC, Akaike information criterion; AUC, area under the receiver operating characteristic curve; LAR, lactate dehydrogenase-to-albumin ratio.

## Data Availability

The datasets generated and/or analyzed during the present study are not publicly available but may be made available by the corresponding author upon reasonable request, subject to institutional review and approval by the Department of Clinical Oncology, University of Health Sciences Antalya Training and Research Hospital.

## Abbreviations

The following abbreviations are used in this manuscript:

AIC	Akaike information criterion
ATRX	alpha-thalassemia/mental retardation syndrome X-linked
CAR	C-reactive protein-to-albumin ratio
CNS	central nervous system
CR	complete response
CRP	C-reactive protein
ECOG	Eastern Cooperative Oncology Group
IDH	isocitrate dehydrogenase
IPCW	inverse probability of censoring weighting
LAR	lactate dehydrogenase-to-albumin ratio
LDH	lactate dehydrogenase
MAR	monocyte-to-albumin ratio
MGMT	O6-methylguanine-DNA methyltransferase
NLR	neutrophil-to-lymphocyte ratio
OS	overall survival
PD	progressive disease
PFS	progression-free survival
PIV	pan-immune-inflammation value
RANO	Response Assessment in Neuro-Oncology
SII	systemic immune-inflammation index
SIRI	systemic inflammation response index
TMZ	temozolomide
WHO	World Health Organization
WHO CNS5	2021 World Health Organization Classification of Tumours of the Central Nervous System
